# Flavonoids as Natural Anti-Inflammatory Agents in the Atopic Dermatitis Treatment

**DOI:** 10.3390/pharmaceutics17020261

**Published:** 2025-02-15

**Authors:** Nurul Ain Zawawi, Haslina Ahmad, Rajesh Madatheri, Nur Izzah Md Fadilah, Manira Maarof, Mh Busra Fauzi

**Affiliations:** 1 Department of Tissue Engineering & Regenerative Medicine, Faculty of Medicine, Universiti Kebangsaan Malaysia, Cheras, Kuala Lumpur 56000, Malaysia; p143394@siswa.ukm.edu.my (N.A.Z.); izzahfadilah@ukm.edu.my (N.I.M.F.); manira@ukm.edu.my (M.M.); 2 Department of Chemistry, Faculty of Science, Universiti Putra Malaysia, Serdang 43400, Selangor, Malaysia; haslina_ahmad@upm.edu.my; 3 Integrated Chemical Biophysics Research, Universiti Putra Malaysia, Serdang 43400, Selangor, Malaysia; 4 Zitai Regeneration Cell Sdn Bhd, George Town 10200, Pulau Pinang, Malaysia; madatheri.rajesh@gmail.com; 5 Advance Bioactive Materials-Cells UKM Research Group, Universiti Kebangsaan Malaysia, Bangi 43600, Selangor, Malaysia; 6 Ageing and Degenerative Disease UKM Research Group, Universiti Kebangsaan Malaysia, Bangi 43600, Selangor, Malaysia; 7 Pharmaceuticals and Pharmacy Practice UKM Research Group, Universiti Kebangsaan Malaysia, Bangi 43600, Selangor, Malaysia

**Keywords:** autoimmune, flavonoids, atopic dermatitis, phytochemicals, polyphenolics

## Abstract

Eczema is a complex autoimmune condition characterised mainly by inflammation and skin lesions along with physical and psychological comorbidities. Although there have been significant advances in understanding the mechanisms behind atopic dermatitis, conventionally available treatments yield inconsistent results and have some unintended consequences. In today’s digital age, where knowledge is just a click away, natural-based supplements have been on the rise for a more “natural” treatment towards any type of disease. Natural compounds, particularly derived from medicinal plants, have piqued significant interest in the development of herbal remedies for chronic inflammatory skin conditions. Among many compounds, flavonoids have shown promise in treating eczema due to their strong anti-inflammatory, antioxidant, and anti-allergic properties, making them helpful in preventing allergic reactions, inflammation, and skin irritation. This review highlights the therapeutic potential of flavonoid-based bioactive compounds to manage eczema, emphasising the mechanisms of action. Additionally, providing a comprehensive analysis of the potential of emerging and established compounds, while bridging a gap between traditional and modern medicine. Flavonoids offer a variety of opportunities for further research and innovative formulations that can maximise its full benefits. Further combination of flavonoids with various approaches such as nanoencapsulation for enhanced bioavailability, hydrogel-based delivery systems for a controlled release, and additive manufacturing for personalised topical formulations, could align with future precision medicine needs.

## 1. Introduction

### 1.1. Overview of Atopic Dermatitis

**Figure 1:**
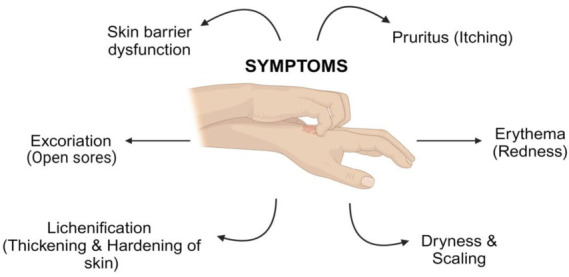
Key symptoms are associated with atopic dermatitis, a chronic inflammatory skin condition.

Atopic dermatitis (AD), commonly known as eczema, once thought to affect children primarily, is now recognised as a condition that also commonly impacts adults, with an estimated prevalence of 3–10% in the general population [1]. Atopic dermatitis is a non-infectious and prolonged chronic inflammatory disorder distinguished by its dry, irritated, pruritic, and eczematous lesions typically located on flexural surfaces (i.e., backs of the knees, inner elbows, and wrists) (Figure 1) [2]. Its known mechanism involves a combination of immune dysregulation, microbial dysbiosis, and a dysfunctional skin barrier. It is typically triggered by various environmental, physiological, and psychological factors, collectively leading to a heightened inflammatory response, which can result in allergic sensitisation [3,4].

**Figure 2:**
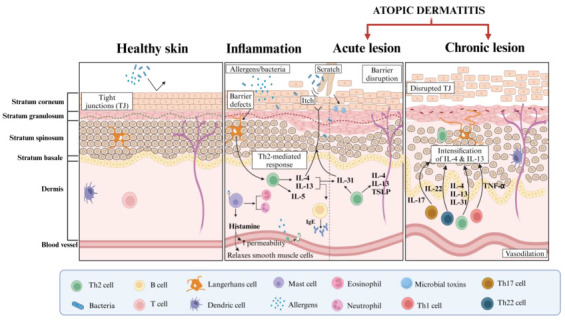
Progression of skin barrier disruption and a focus on type 2 inflammation in atopic dermatitis (AD). The left panel illustrates normal skin with intact tight junctions (TJs) and a balanced immune environment, preventing the entry of allergens and bacteria. In the middle panel, it depicts subcutaneous inflammation and acute AD, the introduction of allergens and pathogens, in conjunction with mechanical disruption through scratching, compromises the barrier function and the release of Th2 cytokines (IL-4, IL-5, IL-13), which facilitates the recruitment of immune cells and promote inflammation. Histamine release increases the vascular permeability of the blood vessel, allowing immune cells to infiltrate the skin and contributing to further inflammation and barrier breakdown. The right panel depicts chronic AD, where repeated barrier disruption leads to an increase in Th2 and Th1 cytokine release (IL-17, IL-22, IL-31), causing further inflammation, epidermal hyperplasia, and fibrosis. Chronic exposure to microbial toxins and allergens exacerbates the immune response, with Th22 and Th17 cells playing a prominent role in chronic lesions, leading to continuous tissue damage and thickened, leathery skin.

The skin barrier, composed of stratum corneum and tight junctions, works together to prevent transepidermal water loss (TEWL) and restrict the entry of microbes, allergens, and irritants. Epidermal barrier components such as filaggrin, loricrin, keratin filaments, lipids, corneodesosin, and kallikreins support this process [5]. The filaggrin (FLG) gene found within the stratum corneum is crucial for maintaining the skin barrier. Its degradation reduces the integrity of the stratum corneum and alters the skin’s acidity and hydration [6]. FLG deficiency can be caused by genetic predispositions or external factors like changes in environmental humidity and air pollution. However, for those affected with atopic dermatitis, the impairment of the skin barrier allows the entry of external antigens, triggering a pro-inflammatory response and increasing the skin’s permeability (Figure 2) [7,8]. This can lead to the colonisation of a bacteria known as *Staphylococcus aureus*, which further enhances the inflammatory response by infecting the lesional and non-lesional areas of the skin in atopic dermatitis patients [9]. This is because *S. aureus* produces toxins and enzymes that damage the skin tissues of patients and act as superantigens that penetrate the skin barrier and induce chronic inflammation [10]. The interaction of superantigens with the immune system can lead to an increase in the production of Th2 cytokines, contributing to chronic inflammation and skin barrier dysfunction [11]. This interplay highlights the role of immune dysregulation in atopic dermatitis, particularly the contribution of Th2-driven pathways in amplifying inflammation and skin damage.

### 1.2. Role of Inflammation in Atopic Dermatitis

Atopic dermatitis involves several immune pathways, such as T helper (Th) 1, 2, 17, and 22 cell activation pathways. It is a T cell-mediated inflammatory disease caused by immune dysregulation and skin barrier dysfunction. Defects in keratinocyte differentiation led to a compromised stratum corneum. This allows the penetration of allergens and microbes, stimulating Immunoglobin (Ig)E sensitisation and type 2 cytokine production, resulting in allergic inflammation [12]. While other pathways play a vital role in the development of eczema, the Th2 pathway is particularly prominent, especially in the acute phase of the disorder. In the Th2 pathway, T cells play an important role in the immune regulation and pathogenesis of the disorder. Upon stimulation by antigen-presenting Langerhans cells, naïve T helper cells will differentiate into Th2 effector cells, which secretes cytokines responsible for the hallmark inflammatory symptoms, encompassing swelling, erythema, and skin irritation [13]. The Th2 pathway has been extensively discussed in its association with the overexpression of cytokines interleukin (IL)-4, IL-5, IL-13, and IL-31 (Figure 2). It also activates various signalling pathways, especially the JAK/STAT pathway, crucial for the pro-inflammatory effects and epidermal barrier disruption [14]. These cytokines, secreted by Th2 cells and other immune cells, contribute to the sensitisation of sensory nerves, which penetrate the tight junctions in the areas of epidermal disruption [8,15,16].

This sensitisation decreases the itch threshold, leaving the skin vulnerable to itching from mechanical and chemical stimulation [15]. Consequently, a continuous and vicious cycle, known as the itch–scratch cycle, is formed between inflammation and the skin barrier. In addition to T cells, innate immune cells like mast cells, eosinophils, basophils, and inflammatory dendritic cells contribute to pathology. Mast cell degranulation releases histamines and promotes chronic allergic reactions, while dysregulation of innate lymphoid cells (ILC2s) exacerbates inflammation [5].

In acute atopic dermatitis lesions, there is robust activation of Th2, Th22, and Th17 cells, whereas as the disorder progresses into the chronic phase, the T cells further polarise into Th1 effector cells [13]. This produces cytokines like IL-12, IFN-γ, and TNF-α, contributing to tissue remodelling and persistent IFN-γ-driven inflammation. T cells, particularly the CLA+ memory T cells, recirculate between skin and peripheral tissues, contributing to persistent inflammation [12]. Other immune cells, including innate lymphoid cells (ILCs), mast cells, eosinophils, and inflammatory dendritic cells, are also present in eczematous lesions. Dysregulated ILCs are stimulated by alarmins like thymic stromal lymphopoietin (TSLP), IL-25, and IL-33, further amplifying the type 2 immune response [8]. The type 2 cytokines, such as IL-4 and IL-13, then suppress the expression of crucial epidermal proteins like filaggrin, loricrin, and involucrin, exacerbating barrier dysfunction [5]. Given the role of T cells in atopic dermatitis, targeting T cell activation has emerged as a potential therapeutic strategy in hopes of preventing the disorder’s progression.

The cytokine-induced disruption of the skin barrier perpetuates allergic inflammation and facilitates the recruitment of additional immune cells, such as eosinophils, basophils, and mast cells, which release mediators like histamine, further contributing to the diseases. This disorder involves multiple complex pathways that increase as the condition worsens, highlighting the need and challenge of finding a multifaceted treatment that can address all aspects of inflammation and break the cycle entirely or provide long-term relief.

### 1.3. Limitations of Current Therapies

Despite the significant advances in science and technology, there has yet to be a definite cure for atopic dermatitis. This chronic skin condition, characterised by its chronic inflammation, itching, and discomfort, continues to challenge medical professionals and researchers. Fortunately, ongoing research into the disorder’s pathophysiology opens the pathway for novel therapies, such as biologics that target specific immune pathways, phototherapy, calcineurin inhibitors, or Janus kinase (JAK) inhibitors. While current treatments often provide symptomatic relief, they may fail to fully address the complexity of atopic dermatitis, especially for patients with a more severe or chronic condition. Moreover, conventional treatments are often associated with limitations, side effects, and concerns about their safety and effectiveness, especially in long-term management. A detailed summary of the mode of delivery, mechanism of action, and the adverse effects caused by the use of conventional therapies are given in Table 1. This has led to an interest in exploring natural-based therapies as a potentially safer solution that could produce similar or superior effects with minimal to no side effects [17].

## 2. Plant-Based Bioactive Compounds

Plant-based foods derived from natural or modified plant sources provide essential nutrients and bioactive compounds that enhance health and prevent diseases [58]. Bioactive compounds, specifically secondary metabolites, play a significant role due to their antioxidative, anti-ageing, and anti-cancer properties [59]. Fruits, vegetables, leaves, roots, flowers, and grains are excellent sources and examples of diversified bioactive compounds found in the different groups of phytochemicals [60]. Among the bioactive compounds, the most extensively researched groups for anti-inflammatory properties are polyphenol and its subgroup of flavonoids, where its wide availability makes it easier to obtain [60]. Therefore, this review aims to summarise current knowledge on the potential of plant-derived bioactive compounds, specifically flavonoids from the group polyphenols, for the prevention and treatment of atopic dermatitis. This review will provide insight into the mechanisms underlying the flavonoid compounds’ potential anti-inflammatory, antioxidant, and immunomodulatory properties.

### 2.1. Overview of Phytochemicals

**Figure 3:**
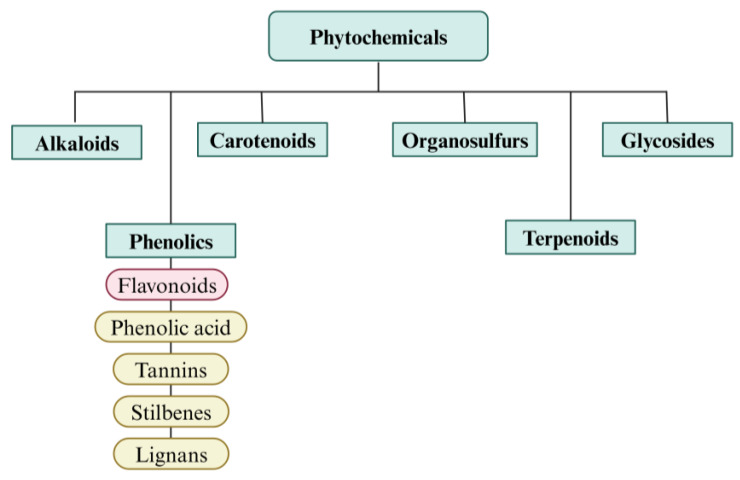
Classification of phytochemicals. Phytochemicals are classified into six categories: alkaloids, phenolics, carotenoids, organosulfurs, terpenoids, and glycosides.

Plants have played a crucial role throughout history, not only as a source of food and oxygen for humans and animals but also for their extensive list of medicinal properties. Plant-based remedies have been used to treat numerous health conditions in all parts of the world. Its incorporation in modern medicine has greatly enhanced the ability to produce a wide variety of bioactive compounds, which opens up new venues for drug discovery and development. Exploring plant-based bioactive compounds underscores a crucial strategy in modern pharmacology, integrating traditional knowledge with cutting-edge science to meet the healthcare challenges of the 21st century. Among these compounds, phytochemicals have captured the attention of many due to their potential health benefits and applications. It has been categorised into groups and subgroups based on its chemical structure and functional qualities, comprising phenolic, alkaloids, terpenoids, glycosides, carotenoids, and many more, as seen in Figure 3. These compounds can be commonly found in fruits, vegetables, legumes, nuts, seeds, and spices [61]. Phytochemicals constitute a large display of chemicals that have been isolated and identified from plants, specifically from their secondary metabolism [62]. Numerous studies have suggested that a diet rich in phytochemicals reduces the risk of cardiovascular and cancer diseases, as well as inflammatory skin conditions as these compounds mainly exhibit antioxidant, anti-inflammatory, and antibacterial properties.

**Figure 4:**
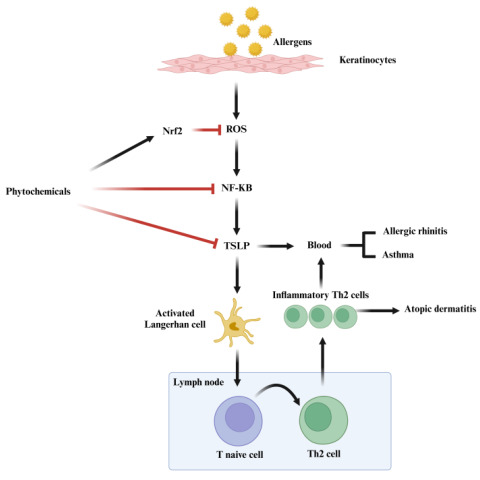
A schematic diagram representing the mechanism of action of phytochemicals in allergic disorders, including atopic dermatitis. The Nrf2 and NF-κB pathways in allergic responses are also represented. Allergens penetrate the skin, activating keratinocytes and Langerhan cells. This leads to the release of ROS (reactive oxygen species) and the activation of NF-κB (Nuclear Factor kappa B), which promotes inflammation and Th2 cell (T helper 2 cell) response, resulting in conditions like allergic rhinitis, asthma, and atopic dermatitis. Phytochemicals can intervene by modulating these pathways.

The mechanism of action of phytochemicals towards allergy disorders can be seen in Figure 4. Firstly, exposure towards allergens triggers the formation of reactive oxygen species (ROS) in keratinocytes, resulting in oxidative stress, which activates the NF-κB signalling pathway. This triggers the release of TSLP, promoting the activation of Langerhans cells (LCs). The activated LCs will then migrate into the lymph node and transfer its antigen to the naïve T cells, leading to the differentiation of T cells into inflammatory Th2 cells. These Th2 cells contribute to the chronic inflammation seen in atopic dermatitis, which can later lead to allergic rhinitis and asthma if prolonged. Phytochemicals have the ability to play a big role by inhibiting the NF-κB and MAPK signalling pathways. It reduces the ROS levels via Nrf2 activation by inhibiting the NF-κB signalling pathway. This reduces the chances of producing pro-inflammatory responses such as TSLP, hence alleviating the inflammatory responses in atopic dermatitis [17]. An overall view of the key classifications of phytochemicals is provided in Table 2, with representative examples for each category. Of all the compounds mentioned, polyphenolics are the most studied and widely distributed group of phytochemicals for anti-inflammatory diseases [62,63].

### 2.2. Polyphenolics and Its Relation to Atopic Dermatitis

Polyphenols, also referred to as phenolics, house different subclasses and are abundant in anti-inflammatory, antioxidant, and antibacterial properties, further contributing to their therapeutic potential across various diseases, including cardiovascular disease, cancer, and skin disorders. It also has over 8000 phenolic compounds and is synthesised by shikimic and malonic acid pathways [107]. Its chemical structure is defined by an aromatic ring that contains one or more hydroxyl substituents characterised into several classes, mainly flavonoids, phenolic acids, tannins, stilbenes, and lignans [63].

Given the extensive benefits, polyphenols exhibit several roles in alleviating the symptoms of atopic dermatitis. Primarily, polyphenols function as antioxidants by obstructing lipid oxidation, reducing hydroperoxide levels, inhibiting the production of reactive oxygen species through enzyme suppression, promoting the synthesis of antioxidant enzymes, regenerating alpha-tocopherol and ascorbic acid, and modulating the signalling pathways of the antioxidant defence system [108]. Furthermore, polyphenols can also stabilise free radicals and effectively diminish their reactivity [62]. A study indicated that administering polyphenols can reverse inflammatory responses, whether taken via dietary consumption or topical application [109]. Additionally, polyphenols can exhibit inhibitory effects towards T cell production and the synthesis of inflammatory cytokine IL-2. It was also found to inhibit the activity of enterotoxins from staphylococcal bacteria [108]. Enterotoxins are proteins secreted by bacteria that can lead to several immune responses, and this can often be seen in the lesional skin of patients with atopic dermatitis, where it is often colonised by *S. aureus* due to its damaged skin barrier. The colonisation of *S. aureus* exhibits inflammatory responses such as itching and redness, which can eventually lead to chronic flare-ups.

Furthermore, a category within the polyphenolics compound, known as flavonoids, is notable for their abundance in nature, therapeutic benefits, and minimal adverse side effects. The focus on flavonoids is significantly influenced by their ability to alleviate immune dysregulation and skin barrier dysfunction, both of which are essential for the management of atopic dermatitis, making it an attractive topic for targeted therapeutic research. Moreover, it provides insight into the modulation of inflammatory-related disorders like atopic disorders.

## 3. Flavonoids: Overview and Relevance

Flavonoids are amongst the most studied and common groups of plant-derived bioactive compounds recognised for their diverse health benefits. They have more than 4000 plant species available and are found abundantly in common fruits and vegetables, making them very accessible for incorporation into a regular diet [109]. Incorporating a flavonoid-rich food into an individual’s diet can help to enhance overall good health. These compounds can also be administered through various ways, such as oral administration, topical application, intravenous injections, or through advanced biomaterial techniques via hydrogels. However, the most common way to obtain the benefits of flavonoids is by eating your fruits and vegetables. Due to its natural antioxidant, anti-inflammatory, and immune-modulating properties, flavonoids have become an ideal therapeutic potential for chronic conditions.

### 3.1. Overview of Flavonoid

**Figure 5:**
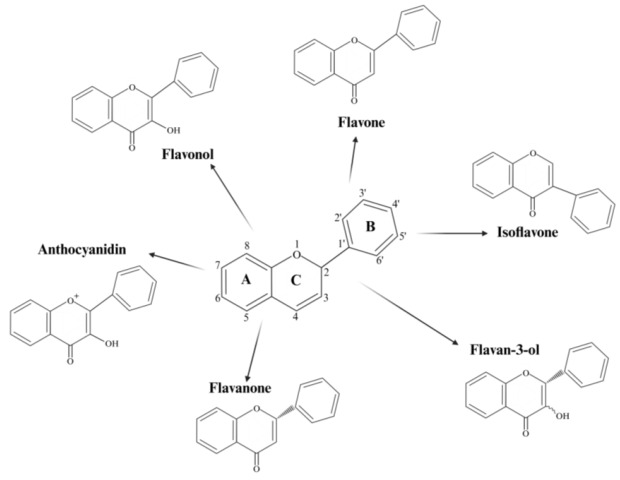
The primary chemical structure of flavonoids and its derivative groups.

Flavonoids are one of the most diverse, studied, identified, and isolated groups of polyphenolic compounds found abundantly in fruits, vegetables, and plant-based foods [110]. Structurally, it is primarily composed of two benzene rings (A and B) connected by a three-carbon bridge to form a heterocyclic pyran ring (C), which forms the basis for various subclasses [111,112]. The subclasses of flavonoids can be further modified depending on the carbon placement of the C ring to the B ring, for example, if the B ring is connected to the third position at the C ring, isoflavones are formed [113,114]. This can be seen further in Figure 5 and Table 3, which shows the primary chemical structure of flavonoids and the different placements of carbon to form flavonols, flavones, flavanones, flavanols, anthocyanidins, and isoflavones, as well as the representatives and source.

### 3.2. Relevance of Flavonoids Towards Atopic Dermatitis

The structural diversity of flavonoids influences biological activities, making them well known for their antioxidant, anti-inflammatory, anti-allergic, immunomodulatory, and antibacterial properties. These attributes highlight flavonoids as suitable candidates for the management of atopic dermatitis, a condition driven by oxidative stress and chronic inflammation. Having said that, the antioxidant properties of flavonoids are especially notable, as oxidative stress plays a key role in keratinocyte damage and an increase in TEWL [120]. The increase in the presence of hydroxyl groups attached to the outermost B ring of flavonoids significantly enhances the antioxidant capacity by effectively neutralising free radicals and ROS while combating oxidative stress [62]. This mechanism is crucial in reducing lipid peroxidation and protein oxidation as well as cellular damage that triggers inflammation and heightens the skin’s vulnerability towards irritants and allergens. Furthermore, flavonoids contribute to skin barrier maintenance by protecting the epidermis from free radical damage, stabilising enzymes like collagen and hyaluronic acid, and improving skin hydration, structure, and resistance towards environmental allergens and irritants [110]. The preservation of these compounds significantly aids pruritus, and irritation and alleviates dryness of the skin.

Beyond their antioxidant capacity, flavonoids also inhibit the secretion of pro-inflammatory mediators and signalling pathways, such as cytokines responsible for inflammation and NF-κB signalling pathway [121]. A previous study identified that certain flavonoids may reduce allergic inflammation by regulating epithelial barriers, indicating its potential therapeutic role in AD [122]. This dual mechanism, which tackles both oxidative stress and regulates inflammation, is what makes flavonoids an attractive and promising approach. Numerous flavonoid extracts have been harnessed to advance eczema treatments and formulations. Compounds such as quercetin and kaempferol have been shown to supress the secretion of IL-4, IL-5, IL-13, eosinophil recruitment, and mast cell activation, all of which are hallmarks of AD. These extracts hold great promise in offering effective solutions for managing and preventing eczema, making them a compelling area of focus for further exploration and development.

## 4. Key Flavonoids in Atopic Dermatitis Treatment

### 4.1. Quercetin

Quercetin, a flavonoid characterised under the group of flavonols, has been extensively studied and exhibited promising results for treating atopic dermatitis over the past few years with its antioxidant and anti-inflammatory properties [113,123]. It is typically extracted from plant sources, such as onions and fruit peels, using solvents like water, ethanol, or acetone [124]. While quercetin is generally considered safe, the dosage typically found over the counter is 500 to 1000 mg daily [125]. Its effectiveness in treating atopic dermatitis is linked to multiple mechanisms. Even though there is no specific pathway, a study demonstrated that quercetin 3-O-2-alpha-L-rhamnopyranoside inhibits the caspase 8 and mitochondrial pathway which allows the prevention of apoptosis of keratinocytes caused by oxidative stress [108]. These actions mitigate the impact of reactive oxygen species (ROS), which is stimulated by NADPH oxidase in inflamed skin, influencing the NF-κB and AP-1 pathways. It can also suppress the activity of pro-inflammatory mediators such as NF-κB and MAPK pathways, which are critical in the inflammatory processes for activating the Nrf2 signalling [108].

Quercetin’s anti-inflammatory efficacy in atopic dermatitis models is further supported by its ability to suppress cytokines like IL-4, IL-5, and TNF-α. A study using an MC903-induced AD mouse model showed that the addition of quercetin significantly reduced the severity of AD and the thickness of the ear epidermis, indicating a reduction in mast cell infiltration and low expression of cytokines such as CCL17, CCL22, IL-6, and IFN-γ [5,62]. In another study, HaCaT (high sensitivity of human epidermal keratinocyte) cells were used, and it was shown that quercetin was able to modulate the expression of long non-coding RNAs, such as lnc-C7orf30-2, which is closely linked to IL-6 and inflammatory processes [126]. Furthermore, quercetin’s regulation of mast cells, keratinocytes, and Th1/Th2 cells contributes to its protective role in atopic dermatitis [62,127]

Additionally, quercetin has also been shown to effectively reduce itchiness and redness in atopic dermatitis by decreasing skin temperature and suppressing pro-inflammatory cytokines such as IL-6 and TNF-α [113,123]. It also inhibits mast cell degranulation and histamine release, critical for allergic responses, through pathways involving TRPV1 channels and histamine H4 receptors [115]. This action decreases intracellular calcium influx, a key process in histamine-induced itching, which quercetin significantly inhibits [115]. Its role in wound healing through promoting epithelial–mesenchymal transition and the upregulation of enzymes like SOD1, catalase, and glutathione peroxidase further solidifies its therapeutic potential [127].

Recent studies have explored quercetin-based formulations for managing AD. Quercevita^®^ 1% cream is a topical formulation characterised by a phospholipids-based delivery system due to its poor solubility and low skin permeability. It is one of the many topical creams that have been evaluated in a single-blind clinical trial, showing a significant decrease in itching sensation and an increase in skin hydration [128]. However, there is still a long way to go, and although quercetin exhibits a lot of benefits, advancements in formulation strategies are needed to ensure patients can fully harness the therapeutic benefits of quercetin. Due to its limited penetration through the skin barrier, other potential delivery methods have been explored, such as combining quercetin with penetration enhancers like liposomes for a transdermal administration of drugs [129] or using encapsulation techniques like incorporating quercetin into a hydrogel to improve stability [130]. Additionally, lipid-based nanoparticles have also been explored to enhance quercetin’s skin permeation. For example, quercetin loaded into lipid nanoparticles that were prepared using a high-pressure homogenisation have demonstrated an improved skin penetration in vivo and can be a potential carrier for quercetin [131].

Quercetin demonstrates a multi-faceted approach to controlling atopic dermatitis symptoms by acting on oxidative stress, inflammatory pathways, cytokine production, and mast cell activity, making it a promising natural compound for therapeutic use. Its ability to regulate pathways such as NF-κB, MAPK, and Nrf2, alongside its impact on mast cells and histamine responses, presents a compelling case for its inclusion in anti-dermatitis treatments.

### 4.2. Epigallocatechin Gallate (EGCG)

Epigallocatechin Gallate (EGCG), a major polyphenolic compound under the category of flavone-3-ols, is commonly found in green tea leaves extracted using hot water or organic solvents. It is widely used in traditional medicine and doses of more than 1 to 3 g per day is considered safe [132]. However, some studies state that higher doses of EGCG can lead to hepatoxicity in both animals and human [133]. EGCG has shown promising potential in treating and managing atopic dermatitis due to its anti-inflammatory, antioxidant, and immunomodulatory properties [134]. Studies have demonstrated that EGCG can prevent mitigating inflammation within the skin by inhibiting the attachment of CD11b on the surface of circulating T and B lymphocytes [108]. By inhibiting this binding process, EGCG effectively stops these immune cells from migrating to areas of the skin experiencing inflammation. This is further supported by its ability to suppress histamine release from mast cells and basophils, which reduces the likelihood of Immunoglobin (Ig) E-mediated allergic reactions [108]. EGCG has also been shown to inhibit the upregulation of vascular endothelial growth factor (VEGF) and IL-8 in keratinocytes stimulated by tumour necrosis factor (TNF) [135]. The use of topical applications has also been observed to significantly reduce skin inflammation, ear thickness, and immune cell infiltration. This also decreases the macrophage migration inhibitory factor (MIF) and Th1-related cytokines such as TNF-α, IFN-γ, and IL-2, restoring the Th1/Th2 balance in AD-like skin lesions [5]. Clinical trials have also demonstrated that a dosage of 660 μmol/L of EGCG solution was able to significantly reduce the symptoms of breast cancer patients with radiation-induced dermatitis [136].

However, despite the promising therapeutic potential of EGCG, there are a few limitations to this compound. A study discussed the compound’s low stability and bioavailability, which causes the effectiveness of the results to become inconclusive due to poor gastrointestinal absorption and rapid metabolism [137]. The compound’s hydrophilic nature limits the penetration of EGCG into the stratum corneum even when applied topically [138]. To overcome these limitations, researchers are exploring different delivery methods to increase the potential of EGCG. A study explored on the transdermal approach through microneedles and nanoparticles with a once-a-week dose regimen and have demonstrated an improvement in stability and skin penetration via atopic-induced Nc/Nga mouse model. The encapsulated EGCG was said to be able to remain in the dermis for 6 days under a sustained release [138].

Other than that, EGCG is also known to be chemically unstable; however, its epimer, gallocatechin gallate (GCG), has shown similar protective effects against UVB-induced skin damage which interestingly improves the skin elasticity and collagen fibres [139]. EGCG also extends the replicative lifespan of fibroblasts by reducing reactive oxygen species (ROS) and inflammation, decreasing the expression of pro-inflammatory factors such as TNF-α and IL-6 while enhancing antioxidant enzymes like superoxide dismutase (SOD) [140]. These antioxidant and anti-inflammatory effects are crucial in mitigating the chronic inflammation seen in atopic dermatitis skin.

### 4.3. Chrysin

Chrysin is categorised under the group flavones that are naturally found in honey, propolis and fruits, and extracted using solvents such as ethanol or methanol [141]. It is rich in bioactive compounds, such as anti-inflammatory, immunosuppressive, and antioxidant properties. The recommended daily dosage of chrysin is 0.5 to 3 g; however, a higher intake may initiate toxicity [142]. In vivo studies were conducted to demonstrate chrysins’ immunosuppressive effects by inhibiting Th1, Th2, and Th22 responses and the slight reduction in CCL17- and CCL22-mediated Th2 infiltration. In a previous study, researchers induced atopic dermatitis in BALB/c mice using 2,4-dinitrochlorobenzene (DNCB) and house dust mite extracts. The study expressed significant results by suppressing immune responses related to Th1, Th2, Th17, and Th22, as well as decreasing the infiltration of Th2 cells mediated by CCL17 and CCL22 through the p38 MAPK, NF-κB, and STAT1 pathways [62,143]. These effects were observed in both human and primary mouse keratinocytes, demonstrating immunosuppressive effects. The study also found that chrysin was able to decrease IL-33 levels, indicating its potential for treating atopic dermatitis. In another study, chrysin was found to inhibit mast cell infiltration and histamine release, a hallmark of allergic inflammation, by modulating intracellular calcium levels and downregulating pro-inflammatory cytokines such as TNF-α, IL-1β, IL-4, and IL-6, which were dependent on NF-κB and caspase-1 signalling pathways [5]. Additionally, chrysin also can regulate thymic stromal lymphopoietin (TSLP) expression in keratinocytes. By downregulating a transcription factor known as EGR1, chrysin can effectively suppress TSLP expression in atopic dermatitis-like inflammatory conditions [144]. Further molecular studies have revealed that chrysin binds to the ATP-binding pocket of the inhibitor of κB kinase (IKK), thereby preventing IκB degradation and NF-κB activation. This leads to reduced expression of CCL5 and mast cell infiltration [5,145]. These effects were confirmed in vivo, where the oral administration of chrysin alleviated AD-like symptoms by reducing ear thickness and serum histamine levels, inhibiting mast cell infiltration, and reducing the inflammatory responses of Th1, Th2, and Th17 cells [62].

While these preclinical findings are promising, chrysin holds similar limitations as the rest of the flavonoids, such as poor bioavailability due to a low water solubility of 0.005 mg/mL which limits its application [146]. Additionally, clinical studies for chrysin are also quite limited. Hence, to address these challenges, ongoing research is focused on enhancing chrysin’s solubility and bioavailability through advanced delivery methods. One promising solution is by encapsulating chrysin in a microemulsion. For instance, a study formulated a chrysin-loaded microemulsion using Labrasol^®^ as a surfactant, isopropyl myristate as the oil phase, and water as the aqueous phase. This formulation resulted in a transparent, oil-in-water microemulsion with an average droplet size of approximately 74.4 nm and a negative zeta potential, indicating good stability [146].

Although research and clinical trials on the application of chrysin in atopic dermatitis are limited, existing studies do highlight its potential as an emerging compound for future therapies. Its multi-targeted mechanism of action focuses on restoring skin barrier integrity and modulating complex immune responses, not only alleviating the symptoms but also improving patient outcomes significantly.

### 4.4. Kaempferol

Kaempferol is a natural flavonol typically found in tea, broccoli, beans, and more. Typically, a high percentage of solvents like methanol or ethanol are used for extraction, and ultrasound-assisted extraction (UAE) is a widely used method. It has a wide range of pharmacological benefits, including antioxidant, anti-inflammatory, anti-allergy, and antitumor properties, and has been shown to effectively treat inflammatory and autoimmune skin diseases [114]. In terms of atopic dermatitis, it has been proven through oral administration via BALB/c mice that kaempferol exhibits significant potential in the modulation of T-cell activation, which is a necessary process in the treatment of atopic dermatitis [13]. A recent study showed a significant decrease in the expression of CD69, an early marker for T cell activation, and a low synthesis of inflammatory cytokines. Thanks to the direct binding of kaempferol and multidrug resistance-associated protein 1 (MRP-1), which acted as a competitive inhibitor, the transporting activity of kaempferol was suppressed, and T-cell activation was regulated [13].

At a molecular level, kaempferol has also demonstrated its ability to reduce reactive oxygen species (ROS) in normal human dermal fibroblasts (NHDF), inhibit the phosphorylation of key signalling proteins, like JNK and NF-κB, proving its potential as a therapeutic agent against skin inflammation and damage [147]. Aside from that, kaempferol demonstrated a significant reduction in TEWL value on days 5 to 7 of treatment in a study employing a mouse model of skin inflammation similar to atopic dermatitis induced by MC903. This suggests that the compound may inhibit the development of lesions in atopic dermatitis. Additionally, the same study also showed that it was able to suppress TSLP expression, increase the expression of barrier proteins, decrease IL-4/IL-13 expressions in CD4 T cells, and suppress oxidative cells [148]. Kaempferol, however, is known to be a refractory drug, where the drug is resistant to a standard formulation or delivery method. This is why previous studies explored ways to improve the solubility and stability of kaempferol. A previous study combined kaempferol with liposomes and loaded it into a hydrogel formulation which demonstrated good homogeneity and stability during the preparation process, indicating its potential for reliable application [149]. However, its high viscosity may affect the uniformity of the application. Despite this, the in vivo results on 5% DNCB-induced mice showing acute eczema revealed that the hydrogel was rapidly absorbed into the skin upon application, providing sustained drug release over an extended period. A similar study incorporating kaempferol into a carboxymethyl chitosan-based hydrogel (CBP) also shows strong antioxidant activities in in vitro and in vivo studies, as well as exhibiting a higher drug release rate at the pH of 7.4 which may be beneficial towards the skin of atopic dermatitis patients as their skin tends to be more alkaline than healthy skin, potentially releasing more drugs at affected areas for a more targeted drug delivery [150].

Overall, kaempferol’s diverse biological activities make it a compelling candidate for further research and development in the treatment of atopic dermatitis and other autoimmune disorders.

### 4.5. Potential Flavonoid: Ocaline™ PF

Ocaline™ PF is an emerging commercially available product that is a synergistic blend of marine spring water and *Cucurbita pepo* seeds [151]. This formulation was said to inhibit the release of substance P, which is a neuropeptide that interacts with keratinocytes and stimulates them to produce an abundance of inflammatory signals like IL-8 and TNF-α. Inflammatory neurotransmitters bind to the receptors of the skin and trigger the modulation of cell properties, influencing how the cells behave and function. This has an impact on the skin’s natural functions, including immunological responses, cell development, and specialisation [152,153]. However, although it is an emerging commercially available product, research on Ocaline™ PF remains very limited despite its promising potential.

Therefore, this section will focus on the seeds of *C. pepo*, which contain strong antioxidant, antimicrobial, and anti-inflammatory compounds. It is commonly sourced from pumpkin peel, flesh, and seeds, representing a valuable source of total phenolic compounds, flavonoids, and mineral constituents [154]. The yellow to dark orange pigmentation of *Cucurbita* sp. fruits is attributed to their elevated carotenoid content, encompassing carotene, lutein, and zeaxanthin. These pigments function to absorb ultraviolet radiation and blue light in addition to scavenging free radicals and reactive oxygen species (ROS) [155]. Additionally, a study stated that the essential fatty acids in pumpkin seeds help to support skin barrier function and reduce inflammation, which further supports its potential use in managing symptoms [156]. Another study stated that the pumpkin seed extracts were also able to reduce inflammation significantly, reducing the inflammation and oxidative changes in a similar condition as atopic dermatitis. The combination of oral and topical application of pumpkin seed extract was able to mediate the downregulation of pro-inflammatory cytokines, including TNF-α, IL-6, COX-2, and iNOS, alongside the upregulation of antioxidant mechanisms within the dermal and serum environments.

Currently, there is limited research on the effects of C. pepo seed extracts on atopic dermatitis, however, the general anti-inflammatory and antioxidant of properties of these flavonoids suggest potential benefits. Further research is needed to evaluate the effects towards atopic dermatitis. Exploration of other plant extracts that can alleviate the symptoms of atopic dermatitis are also necessary.

### 4.6. Other Flavonoids

In addition to the previously discussed compounds, several other compounds, each with their own unique properties, have been explored for their therapeutic potential in the management of AD. For instance, naringenin, a flavonoid found in citrus fruits, has been shown to inhibit genes activated by NF-κB. A study explored naringenin’s anti-inflammatory and antioxidant benefits by exposing HDFs with lipopolysaccharides to induce skin inflammation. The compound not only reduced the expression of inflammatory cytokines, but it also decreased the inducible nitric oxide synthase (iNOS) expression, making it a compound that shows promising benefits for treating inflammatory skin conditions [157]. Another flavonoid of interest is luteolin, which explored the effects of topical luteolin on BALB/c mice that were induced with atopic dermatitis through the topical application of 2,4-dinitrochlorobenzene (DNCB). The study observed that the treatment of topical luteolin treatment was able to effectively reduce the secretion of Th2 and Th17-related cytokines. It was also observed that the level of Th1-type cytokine in dorsal skin elevated and restored the Th1/Th2 immune balance in AD [158]. The key findings and summary of the natural compounds are further detailed in Table 4.

## 5. Conclusions

In conclusion, atopic dermatitis significantly impacts the quality of life of patients with the disorder. Therefore, finding an effective targeted treatment with minimal side effects is crucial. Flavonoids, a prominent group of polyphenolic compounds derived from naturally occurring compounds, have emerged as a potential therapeutic agent due to their numerous health benefits, such as anti-inflammatory, antioxidant, and anti-allergic properties. Flavonoids have been proven to reduce oxidative stress, modulate immune response, and promote skin repair. While flavonoids have demonstrated their effectiveness in in vitro and in vivo models, large-scale clinical trials are needed to confirm the effectiveness in treating atopic dermatitis. These trials should focus on determining the appropriate dosage, long-term safety, and potential side effects for future expenditure of treatments. Incorporating flavonoid-rich compounds into existing topical treatments, dietary interventions, and wound care dressings, such as topical corticosteroids and more, could enhance the management of chronic skin conditions and lead to a more effective treatment strategy. Continued exploration of these natural compounds could lead to significant advancements in dermatological therapies, positioning flavonoids as a valuable disease prevention and treatment tool.

## Figures and Tables

**Table 1:** Current treatments, their mechanism of action, and their side effects.

Mode of Delivery	Types	Example	Mechanism	Adverse Effects	Refs.
Topical	Emollients and moisturisers	Paraffin cream, Glycerol cream	Hydrating and barrier repair of the skin, reducing water loss, and protecting the skin barrier	Irritation and allergic reactions, folliculitis, acne, stinging, and inconsistent results	[18,19,20,21]
	Topical corticosteroids	Hydrocortisone	Reduce inflammation, suppress the immune system and reduce itching	Skin thinning, striae formation, adrenal suppression, skin atrophy, striae, telangiectasia, rosacea, hormonal imbalances, and haematological changes	[22,23,24,25,26]
	Calcineurin inhibitor	Pimecrolimus	Inhibit calcineurin, reduce T-cell activation and inflammation	Rosacea-like eruptions, skin burning, phototoxic, warmth, and mild pain or stinging at the site of application	[22,27,28,29,30]
		Tacrolimus		Rosacea-like eruptions, burning, stinging, and itching at the application site, and long-term use cause nephrotoxicity and hypertension	[31,32,33]
	Topical phosphodiesterase-4 (PDE4) inhibitors	Crisaborole	cAMP modulation by inhibiting PDE4 and inhibiting basophil activation to reduce inflammation	Burning or stinging sensations, pruritus, and erythema	[34,35,36,37]
Oral	Janus kinase (JAK) inhibitors	Upadacitnib	Inhibits the JAK-STAT signalling pathway, which is involved in the signalling of multiple pro-inflammatory cytokines	Risk of infection, acne, long-term use cases, malignancy, and cardiovascular risks	[38,39,40,41]
		Abrocitinib	JAK1 inhibition and cytokine modulation	Nausea, headache, acne, upper respiratory tract infections, and long-term use cause herpes zoster infections and venous thromboembolism.	[42,43,44,45]
Subcutaneous Injection	Biologics	Dupilumab	Targeting IL-4 and IL-13 pathways	Erythema nodosum, Ocular issues, eosinophilia and hypereosinophilia, skin disorders and musculoskeletal issues	[46,47,48,49,50]
		Tralokinumab	Targeting IL-13 pathway	Upper respiratory tract infections, conjunctivitis, erythema, haematoma	[51,52,53]
Phototherapy	Ultraviolet B (UVB) Phototherapy	Narrowband UVB (NB-UVB) therapy	Reduces T-cell activity, induces apoptosis in skin cells, decreases pro-inflammatory cytokines, reduces *Staphylococcus aureus* colonisation	Potential for post-UVA burns, Xerosis, pruritus, erythema, skin cancer	[54,55,56,57]

**Table 2:** Summary of different phytochemicals and their effects on various diseases, along with the benefits they provide for those conditions.

Classification	Sub-Classification	Compound	Source	Prevention and Treatment of Diseases	Benefits	Refs.
Alkaloids	-	Berberine	Isoquinoline alkaloid and *Berberis aristata* root bark	Lung cancer, metabolic disorders, cardiovascular diseases	Lung cancer, metabolic disorders, cardiovascular diseases	[64,65,66,67,68]
Phenolics	Flavonoids	Quercetin	Onions, apples, *Pulicaria jaubertii*	Neurodegenerative diseases, colon cancer, oxidative stress-related diseases, liver diseases	Neurodegenerative diseases, colon cancer, oxidative stress-related diseases, liver diseases	[69,70]
	Phenolic acid	Gallic acid	Tea leaves, gallnuts, berries, apple peels, honey products, grapes,	Liver and cardiovascular diseases and metabolic dysfunction	Liver and cardiovascular diseases and metabolic dysfunction	[71,72,73,74,75]
	Tannins	Tannic acid	Tea, oak bark	Liver disorders, neurodegenerative diseases, inflammatory conditions, viral infections, and cancer	Liver disorders, neurodegenerative diseases, inflammatory conditions, viral infections, and cancer	[76,77,78]
	Stilbenes	Resveratrol	Red wine, grapes	Obesity, cardiovascular diseases, cancer, and neurological disorders	Obesity, cardiovascular diseases, cancer, and neurological disorders	[79,80,81,82,83,84]
	Lignans	Secoisolariciresinol	Flaxseeds, sesame seeds	Hormonal balance disorders, cancer prevention, metabolic disorder, cardiovascular diseases	Hormonal balance disorders, cancer prevention, metabolic disorder, cardiovascular diseases	[85,86,87,88,89]
Terpenoids	-	Ginkgolides	Leaves of the *Ginkgo biloba* tree.	Neurological and cerebrovascular disorders	Neurological and cerebrovascular disorders	[90,91,92,93]
Glycosideds	-	Gracillin	Plants from the Dioscoreaceae family	Cancer, pulmonary fibrosis, and cardiac injury	Cancer, pulmonary fibrosis, and cardiac injury	[94,95,96,97,98]
Carotenoids	-	Β-carotenes	Carrots, sweet potatoes	Eye health (macular degeneration), cancer, inflammation, cardiovascular and viral infections	Eye health (macular degeneration), cancer, inflammation, cardiovascular and viral infections	[99,100,101,102]
Organosulfurs	-	Lipoic acid	Spinach, broccoli	Diabetes management, neurodegenerative diseases, metabolic disorders, and viral infections	Diabetes management, neurodegenerative diseases, metabolic disorders, and viral infections	[103,104,105,106]

**Table 3:** Division of flavonoids and its sources.

Flavonoid Group	Representatives	Primary Food Source	Ref.
Flavonols	Quercetin Kaempferol Myricetin Fisetin Rutin	Lettuce, onion, kale, broccoli, fruit peels, cherries, berries, black tea, red wine, grapes	[115]
Anthocyanidins	Cyanidin Pelargonidin Delphinidin Peonidin Malvidin	Berries, raspberry, pomegranates, tea, fruit peels with dark pigments, eggplant, red cabbage, violet cauliflowers	[116]
Flavonones	Naringenin Naringin Hesperetin Eriodictyol	Citrus, lemon, orange, grapefruit, citrus peels	[115]
Flavone-3-ols	Catechin Epicatechin Gallocatechin Dihydrokaempferol	Tea, apples, pears, berries, chocolate/cocoa products	[117]
Isoflavones	Genistein Daidzein Glycitein	Soy-derived products, alfalfa, beans	[118]
Flavones	Apigenin Luteolin Diosmin Baicalein Chrysin	Parsley, lettuce, thyme, red wine, tomato skin	[119]

**Table 4:** Summary of the natural compounds and its key finding in the in vivo or in vitro model for the treatment of atopic dermatitis.

Flavonoid	Model	Key Findings	Ref.
Quercetin	HaCaT cells	Modulates lncRNA (lnc-C7orf30-2) linked to IL-6.	[126]
	MC903-induced AD mouse model	Reduces ear thickness, mast cell infiltration, and cytokine expression (CCL17, CCL22, IL-6, IFN-γ).	[62]
Epigallocatechin Gallate (EGCG)	Keratinocytes stimulated by TNF, and fibroblasts	Suppresses release of VEGF, IL-8, and histamine.	[135]
	Atopic induced Nc/Nga mouse model	Encapsulation (microneedles/nanoparticles) prolongs dermal retention for 6 days and improves the skin penetration and stability.	[138]
Chrysin	BALB/c mice by the repeated local exposure of 2,4-dinitrochlorobenzene (DNCB) and house dust mite to the ears	Reduces ear thickness and histamine levels, as well as inhibiting Th1, Th2, Th17, and Th22 pathways.	[143]
Kaempferol	Human dermal fibroblast (HDF)	Inhibits phosphorylation of JNK and NF-κB signalling pathway.	[147]
	MC903-induced AD mouse model	Reduces TEWL and improves skin barrier function. Inhibits TSLP, IL-4, IL-13 expressions and oxidative cells in CD4 T cells.	[148]
Naringenin	Human dermal fibroblasts (HDFs) exposed to lipopolysaccharide (LPS) to induce skin inflammation	Significantly reduced the expression of inflammatory cytokines by inhibiting NF-κB activation and was able to decrease inducible nitric oxide synthase (iNOS) expression.	[157]
Luteolin	BALB/c mice induced with DNCB	Regulated the production of Th1/Th2/Th17-mediated cytokines and inhibited the phosphorylation of JAK2 and STAT3 in the lesional skin.	[158]

## Data Availability

The authors confirm that the data supporting the findings of this study are available within the article.

## Abbreviations

The following abbreviations are used in this manuscript:

AD	Atopic dermatitis
TEWL	Transepidermal water loss
Th	T-helper
IL	Interleukin
TSLP	Thymic stromal lymphopoietin
ILCs	Innate lymphoid cells
Th2	T-helper 2 cells
JAK-STAT	Janus Kinase-Signal Transducer and Activator of Transcription
CLA+	Cutaneous Lymphocyte Antigen Positive
NF-kB	Nuclear Factor Kappa-Light-Chain-Enhancer of Activated B Cells
MAPK	Mitogen-Activated Protein Kinase
AP-1	Activator Protein 1
TRPV1	Transient Receptor Potential Vanilloid 1
SOD1	Superoxide Dismutase 1
STAT1	Signal Transducer and Activator of Transcription 1
CD69	Cluster of Differentiation 69
COX-2	Cyclooxygenase-2
iNOS	Inducible Nitric Oxide Synthase
